# Do concepts of individuality account for individuation practices in studies of host–parasite systems? A modeling account of biological individuality

**DOI:** 10.1007/s12064-024-00426-3

**Published:** 2024-09-13

**Authors:** Nina Kranke

**Affiliations:** Chair of Nature Conservation and Landscape Ecology, University of Freiburg, Stefan-Meier-Str. 76, 79104 Freiburg, Germany

**Keywords:** Biological individuality, Individuation practices, Host–parasite interaction, Host–parasite coevolution, Models, Pluralism

## Abstract

In recent discussions, the widespread conviction that scientific individuation practices are governed by theories and concepts of biological individuality has been challenged, particularly by advocates of practice-based approaches. This discussion raises questions about the relationship between individuation practices and concepts of individuality. In this paper, I discuss four studies of host–parasite systems and analyze the respective individuation practices to see whether they correspond to established concepts of biological individuality. My analysis suggests that scientists individuate biological systems on different levels of organization and that the researchers’ respective emphasis on one of the levels depends on the explanandum and research context as well as epistemic aims and purposes. It thus makes sense to use different concepts of individuality to account for different individuation practices. However, not all individuation practices are represented equally well by concepts of biological individuality. To account for this observation, I propose that concepts of individuality should be understood as abstracted, idealized, or simplified models that represent only certain aspects of scientific practice. A modeling account suggests a pluralistic view of concepts of biological individuality that not only allows the coexistence of different kinds of individuality (e.g., evolutionary individuality, immunological individuality, ecological individuality) but also of normative and descriptive concepts.

## Introduction

In discussions of biological individuality, it is often implied that biologists need a clear concept of individuality to successfully carry out empirical work. This conviction is based on at least two interrelated assumptions about the relationship between concepts of individuality and scientific practices. The first assumption is that biologists follow a top-down approach, meaning that they adopt an inner-scientific or meta-scientific^1^ concept of individuality and apply this concept to the systems of interest^2^ (see Clarke 2010, 2013; DiFrisco 2019). The second assumption concerns the character of concepts of individuality. It is believed that concepts of individuality are theories that provide answers to the question “What is an individual?” as well as criteria that determine whether a biological system is an individual or not (Kovaka 2015; Baedke 2019; Kaiser and Trappes 2021; Wilson and Barker 2024). In other words, they are widely understood as prescriptive concepts that govern scientific practices.

With the rise of practice-oriented philosophical work on individuation and individuality, however, the priority of theoretical over empirical and practical aspects of the discussion on individuality has been challenged (e.g., Kendig 2016; Kovaka 2015; Nyhart and Lidgard 2017; McConwell 2023). Karen Kovaka (2015), for example, characterizes the relationship between concepts of individuality and scientific practices as reciprocally dependent. She argues that empirical research is sensitive to existing concepts of individuality, but scientists do not rigorously follow a top-down approach to individuate biological systems. Practice-oriented philosophical studies of biological individuality have also spawned new ways of thinking about scientific practices in relation to meta-scientific approaches. Alan Love (2018) and Kenneth Waters (2018), for example, suggest that philosophers of science should follow a bottom-up approach and study individuation practices in the sciences to understand for what purposes scientists individuate biological systems and how their practices serve their epistemic aims. Thus, instead of asking whether concepts of individuality are suitable for guiding scientific practices (e.g., Sterner 2015), one could ask how well these concepts describe and explain scientific practices. The discussion raises interesting questions about the relationship between concepts of individuality and individuation practices. For example, “What exactly is the role of concepts of individuality, to guide empirical work, to describe and explain scientific practices, or both?”. But the discussion of the relationship between concepts of individuality and individuation practices also relates to questions about the relationship between scientific practices and concept formation in the sciences and philosophy.

In this paper, I explore this relationship by analyzing four studies of host–parasite systems. After describing the scientists’ experimental and theoretical work, I focus on the question of how scientists individuate biological systems. As a third step, I analyze to which concepts of individuality these scientific practices^3^ correspond and discuss whether the concepts adequately capture the practices in question. I conclude that within one research field, scientists individuate similar biological systems on different levels of organization (e.g., cells, organisms, host–parasite systems as a whole). The researchers’ respective emphasis on one of the levels corresponds to their epistemic aims or purposes and the explanans. Thus, it makes sense to use different concepts of individuality to account for different individuation practices. My analysis also shows that individuation practices of some scientific studies can be captured adequately by concepts of individuality but sometimes there is a mismatch between practice and theory. These findings can be explained by a relationship of reciprocal dependence between meta-scientific and inner-scientific concepts of individuality and individuation practices. Does this mean that the concepts need to be revised, or should scientists rethink and adjust their practices to bring them in line with existing concepts of individuality?

While in some cases one of these two options might be justified, I argue that it is not always required to adjust either theory or practice. I argue that concepts of individuality should be understood as different models. Models are not true or false in a general sense, but adequate or inadequate depending on the context of use. As models, concepts of individuality are simplified, abstract, or idealized representations of scientific practice and it cannot be expected that they capture all instances of scientific practice, even within one research field.

## Biological individuality

In this section, I briefly introduce three different meta-scientific conceptions of biological individuality, i.e., immunological, evolutionary and ecological individuality, that are relevant for my analysis of the individuation practices in studies of host–parasite systems in the following sections.

The concept of immunological individuality was proposed by Thomas Pradeu (2012). Immunological individuality is a kind of physiological individuality that applies immunogenicity, i.e., the ability to induce immune responses, as a criterion for individuality. To put it simply, if an entity is rejected by an organism’s immune system, it is not part of that organism (Pradeu 2012, 240). Immunological individuality is a boundary-centered approach where the boundaries are established by an organism’s immune system. To determine whether an entity is part of an organism thus requires knowledge of the organisms’ immune responses, e.g., through isolation and analysis of cells.

Evolutionary individuality can either refer to units of evolution, i.e., biological units that evolve, or units on which natural selection operates (objects of natural selection). Genealogical individuals (units of evolution) are lineages such as species and phylogenetic taxa (Hull 1978). They are historical entities “localized in space and time, individuated spatiotemporally, and made up of spatiotemporally organized parts” (Hull 1976, 177). Just like a human individuals, species or other lineages persist over time although they undergo changes.

Building on Lewontin’s (1970) account of natural selection, Peter Godfrey-Smith (2009) understands evolutionary individuals as units of selection (“Darwinian individuals”). According to Godfrey-Smith (2009, 39) a Darwinian population is “a collection of causally connected individual things in which there is variation in character, which leads to differences in reproductive output (differences in how much or how quickly individuals reproduce), and which is inherited to some extent.” Darwinian individuals are members of such a population. Godfrey Smith’s account thus emphasizes the establishment of parent–offspring lineages and variation, i.e., heritable differences (Wilson and Barker 2024).

To account for the individuality of ecological communities, Philippe Huneman (2014a, b) proposed a concept of individuality that is based on the notion of quasi-independence. In a quasi-independent subsystem, the interactions between elements in the system are stronger than interactions between external elements (Huneman 2014a, 364). His concept of individuality prioritizes interactions over boundaries and the application of the concept produces nested individuals (Huneman 2014b). Ecological individuality is a continuum with strong and weak individuals being differentiated by the connections among their components. It is, however, a formal concept and its application to a system requires a theory to define the variables in question and empirically determine individuality. It can be applied to different types of systems (e.g., cells, organisms, communities) by choosing a relevant theory (Huneman 2014b).

## Individuation practices

In this section, I discuss individuation practices in recent studies of host–parasite systems by means of case studies. Host–parasite systems comprise members of at least two different species that interact in various ways. The question of how researchers individuate biological entities when they study host–parasite systems is particularly interesting because hosts and parasites are often tightly interconnected. In this context, I understand ‘individuation practices’ as the practices that scientists employ to single out a biological system as a distinct entity (see Lowe cited in Bueno et al. 2018, 2). In this paper, I focus on studies of helminths (various species of parasitic worms) and their vertebrate hosts (e.g., fishes, mammals). I discuss scientific practices, particularly experimental research and conceptual practices (e.g., the use and construction of concepts^4^). All four studies are comparable with respect to the organisms that were investigated and to the researchers’ engagement with immunology. I first describe each study and subsequently analyze the respective individuation practices and reconstruct these practices by using meta-scientific concepts of biological individuality. For each study, I focus on the main unit of analysis identified by the researchers.^5^

### Host–parasite interaction

#### Description of the study

The first study was published by Jörn Scharsack and collaborators in 2004 (Scharsack et al. 2004). In the study, the scientists investigated cellular immune responses of three-spined sticklebacks (*Gasterosteus aculeatus*) to infections with tapeworms (*Schistocephalus solidus*). As part of a series of experiments the group infected the fish with tapeworms through ingestion of infected copepods (small crustaceans). As white blood cells indicate an immune reaction to parasites and infections in general, the researchers isolated the sticklebacks’ white blood cells and analyzed them using flow cytometry.^6^ They also measured the parasites’ body weight throughout the observation period of 98 days.

The team found that some of the infected fish had cleared the infection, but others were not able to get rid of the parasite. The analysis of the white blood cells showed that during the initial phase of the infection, proportions of granulocytes increased while proportions of lymphocytes^7^ decreased. This result is not surprising, as the “mobilisation of granulocytes is a common feature of the immune responses of fishes to helminth parasites” (Scharsack et al. 2004, 147). However, after day 63 of the infection, the proportions of granulocytes decreased, while the parasites’ body weight increased continuously throughout the observation period. The researchers thus hypothesize that “this might reflect the ability of *S. solidus* to impair the cellular response of its host” (Scharsack et al. 2004, 147). Based on their results, Scharsack and his collaborators (2004, 148) conclude that the stickleback’s immune response is only effective against the tapeworm until the parasite has researched a certain developmental stage: “The initial phase of infection seems to be decisive for the development of a parasitosis. It is likely that mobilization and activation of granulocytes can be effective against procercoid^8^ stages of *S. solidus* during the first weeks of infection, but once plerocercoid^9^ stages are present in the body cavity, granulocytes are unable to develop their activity against *S. solidus.*”

#### Analysis of individuation practices

For their experiments, the scientists use white blood cells to study the stickleback’s cellular immune response. Both the research question and the experimental setting presuppose the notion of stickleback and tapeworm as individual organisms with an antagonistic relationship. The individuation on the level of the organism precedes the isolation of cells and is at the same time confirmed by the results of the experiment. The focus of this study is on the host and its reaction to the infection with the parasite. The scientists conceptualize the white blood cells as parts of the stickleback’s immune system that reacts to the tapeworm by mobilizing granulocytes.

The individuation of cells and organisms in this experiment corresponds to the concept of immunological individuality proposed by Thomas Pradeu (2012). In the article published by Scharsack et al. (2004), the mobilization and activation of granulocytes are clearly interpreted as an immune reaction of the host to an invader. Since tapeworms trigger immune responses in sticklebacks, they are not a part of the stickleback, but separate individuals. Following Pradeu’s concept of immunological individuality, one could say that the boundaries between stickleback and tapeworm are established by the stickleback’s immune response. Although stickleback and tapeworm were already recognized as individual organisms prior to the experiment, the immunological individuality of the stickleback was confirmed by the analysis.

### How host evolution affects parasites

#### Description of the study

The second case study is an experiment by Jesse Weber et al. (2017) who have also conducted research on the stickleback–tapeworm experimental system. The starting point of the study was the observation that populations of sticklebacks (*Gasterosteus aculeatus*) on Vancouver Island varied significantly in their infection prevalence.^10^ The Gosling Lake (GOS) population, for example, exhibited an infection prevalence of 50–80%, while no tapeworm (*Schistocephalus solidus*) infections were observed in the Roberts Lake (ROB) population. The aim of the group’s research was to test whether these two populations vary with respect to immune phenotypes and to evaluate whether there are underlying genetic differences that explain the variation (Weber et al. 2017). To see whether the variations in infection prevalence is associated with heritable differences in immune response, the team conducted a breeding experiment in the laboratory. Wild-caught stickleback from GOS and ROB was bred and crossed to generate different pure and hybrid populations (Weber et al. 2017, 6576).

As in the experiment discussed in the previous section, the fish were fed infected copepods and the infection frequency (proportion of stickleback with *S. solidus*), infection intensity (abundance of *S. solidus* per fish) and mass of the tapeworms were measured. Interestingly, GOS and ROB stickleback did not differ significantly in the frequency or intensity of laboratory infections; however, the parasites grew dramatically larger in GOS than in ROB stickleback. On average, the mass of tapeworms isolated from GOS stickleback was 34-fold larger than the mass of tapeworms from ROB stickleback. In the hybrid populations (GOS stickleback crossed with ROB stickleback) the tapeworms grew to intermediate size (Weber et al. 2017, 6577). According to Weber et al. (2017, 6577) “these results demonstrate [that] there are heritable and thus evolved differences between the ROB and GOS stickleback, given differences in laboratory-raised sticklebacks’ ability to suppress tapeworm growth. The intermediate size of parasites in hybrid fish also strongly suggests an additive genetic basis for this evolutionary difference.” As growth suppression constrains the tapeworms’ reproductive potential, the greater resistance of ROB stickleback decreases the tapeworm’s fitness (Weber et al. 2007, 6577). Weber et al. (2017, 6577) argue that “this measure of parasite success represents an extended phenotype of the stickleback, in the sense that the host’s genotype alters the parasite’s phenotype.”

#### Analysis of individuation practices

Like the case discussed in the previous section, individuation takes place both prior to the experiment and in the course of evaluating and explaining the results. The way in which the researchers describe the starting point of the experiment and the experimental design shows that the stickleback and the tapeworm are individuated both on the level of the organisms and on the population level. The individuated organisms represent their respective populations (ROB and GOS stickleback). Host and parasite, however, do not evolve completely separately, but their evolutionary trajectories are intertwined (Weber et al. 2017, 6575). Thus, the parasite is not only seen as the host’s immunological antagonist but also as its evolutionary antagonist in the sense that parasite adaptation to the host is followed by host adaptation and so on. This evolutionary scenario where adaptations are followed by counter-adaptations of the opposing species is also known as *Red Queen hypothesis*^11^ (van Valen 1973).

To explain their experimental results, Weber et al. (2017) draw on the concept of the extended phenotype that was introduced by Richard Dawkins (1999 [1982]). The concept of the extended phenotype is closely linked to his concept of the selfish gene. Dawkins (1976) favors a gene-centered view of evolution and understands the gene as the central unit of selection. He argues that the concept of the phenotype should be extended to include “all effects of genes upon the world” (e.g., on the cell, the organisms’ body, artifacts such as spider webs; Dawkins 1999, 293).^12^ In the discussion of their results, Weber et al. (2017) conceptualize the parasite’s growth as part of the host’s phenotype. Following Dawkins, this would mean that the small size of tapeworms from ROB stickleback is an effect of ROB stickleback genes. Weber et al. (2017) only examined whether there is an underlying genetic difference between ROB and GOS stickleback. However, they did not study what exactly the genetic differences are and thus the genes responsible for the repression of tapeworm growth have not been individuated.

In the experiment, the focus is not on the nature of the immunological reaction itself but on the evolutionary interconnectedness of host and parasite. Thus, I explore whether the explanatory practices in question can be captured by concepts of evolutionary individuality. In the literature, evolutionary individuality is usually associated with units of selection (e.g., Godfrey-Smith 2009; Gould and Lloyd 1999; Lewontin 1970). I have argued that Weber and his collaborators view the stickleback and the tapeworm as evolutionarily intertwined. The question is whether the host–parasite system as described by Weber et al. (2017) can be characterized as an evolutionary individual. I argue that this is not the case because the host–parasite system as a whole is not targeted by selection and the host’s fitness is not aligned with the parasite’s fitness (Bourrat and Griffiths 2018; Clarke 2013). It has been argued that multispecies conglomerates can be considered evolutionary individuals. Ereshefsky and Pedroso (2015), for example, claim that multispecies biofilms could be viewed as evolutionary individuals on the basis of certain characteristics such as internal integrity, division of labor, coordination among parts, and heritable adaptive traits. In the study by Weber et al. (2107), the host and the parasite are described as evolutionary antagonists, meaning that an adaptation that benefits the stickleback (e.g., growth suppression of the parasite) decreases the tapeworm’s fitness. The adaptation in question is therefore not located on the level of the host–parasite system as a whole. Although the stickleback–tapeworm system exhibits a certain degree of integrity, there is no division of labor or coordination among parts. Therefore, the system fails to meet at least three of the four criteria suggested by Ereshefsky and Pedroso (2015). On that basis, it seems plausible to argue that the stickleback–tapeworm system as described by Weber and his team is not a unit of selection because host and parasite are conceptualized as antagonists that are not “ultimately ‘in the same boat’” (Bourrat and Griffiths 2018, 33).

To simply reconstruct the researchers’ individuation practices in terms of physiological individuality (stickleback and tapeworm as individual organisms), however, would not adequately capture the close causal relationship between the evolutionary trajectories of stickleback and tapeworm expressed by the use of the concept ‘extended phenotype’ and the description of Red Queen dynamics. Concepts of species or lineages as individuals (e.g., Ghiselin 1974; Hull 1978) could account for the individuation of organisms as representatives of a population, but do not account for the evolutionary interconnectedness of host and parasite. To my knowledge, there is no established meta-scientific concept of biological individuality that accounts for the conceptualization of parasite size as a phenotypic expression of host genes. Or, generally speaking, there is no meta-scientific concept of individuality that captures the “extended phenotype,” a unit that extends beyond an organism’s phenotype and is not a unit of selection.

### The intestinal ecosystem

#### Description of the study

The article discussed in this section is a review by Heather Filyk and Lisa Osborne published in 2016 where they introduce the concept of the multibiome and review literature on multibiome–host interactions and interactions between members of the multibiome. The term ‘multibiome’ is introduced by the scientists “to encompass the diverse collection of microscopic (bacteria, archaea, fungi) and macroscopic (multicellular worms) organisms, as well as viruses […] that colonize mammals” (Filyk and Osborne 2016, 47). The multibiome is comprised of four subsystems, the bacterial microbiome, the virome, the mycobiome and the macrobiome. The difference to the holobiont is that the microbiome does not include the host organism (see following section). The authors discuss the respective systems separately but also highlight the interactions between subsystems and with the host. According to Filyk and Osborne, helminths belong to the microbiome. In the article, the authors discuss several interactions that involve helminths. The reviewed literature suggests, for example, that helminths alter the composition of the bacterial microbiome which can directly influence the host’s immune homeostasis and responses to pathogens (Filyk and Osborne 2016, 49). Helminth-induced immunomodulation can also impair antiviral immunity to newly acquired intestinal viral infection and helminths use commensal bacteria as cues that they have reached their destination to develop (Filyk and Osborne 2016, 49–50).

#### Analysis of individuation practices

Filyk and Osborne (2016, 49) emphasize the interactions between intestinal entities and understand the multibiome as an ecosystem that is regulated by these interactions: “Similar to other ecosystems, the intestinal community is dynamic, responsive, and regulated by interactions between distinct biological entities.” To present the results of their literature review, the authors individuate nested biological systems, e.g., host organism, the entire intestinal ecosystem (multibiome) and subsystems comprised of members of several species such as the macrobiome and the bacterial microbiome. In this study, helminths are conceptualized as constituents of an ecosystem and the host is conceptualized as the environment of the multibiome with which interactions take place. As the multibiome is the central concept in Filyk and Osborne’s article, I will focus my discussion on this concept.

As the multibiome as a whole does not have an immune system, the concept of immunological individuality does not apply. Under a concept of evolutionary individuality, the multibiome or other communities would not be considered an individual because it is not targeted by selection (see Huneman 2014b). The individuation of ecosystems like the multibiome, however, corresponds to Philippe Huneman’s (2014a, b) ecological concept of individuality. The application of the concept produces nested individuals (Huneman 2014b) which corresponds well to Filyk and Osborne’s approach to individuation of nested biological systems. However, the application of the concept to a system requires a theory to define the variables in question and empirically determine individuality. The question of whether the multibiome is an individual is thus an empirical question that cannot be answered on the basis of the information provided by the authors of the review article. However, Huneman (2014b, 376) states that both the host organism and the gut microbiome are individuals under his weak concept of individuality. It is unclear whether his notion of ‘gut microbiome’ also comprises helminths, but with the information in the article by Filyk and Osborne, it is plausible to argue that the multibiome exhibits a higher degree of individuality (in terms of interactions) than a randomly assembled set of organisms and that the individuation of this system based on interactions among members of the system is non-arbitrary.

### Helminths as old friends

#### Description of the study

Like the individuation practices discussed in the previous section, the following case is an instance of individuation in the context of scientific theorizing, not experimental individuation. In this section, I discuss several articles by Graham Rook (2009, 2010, 2012) on a similar topic, the so-called *old friends hypothesis*. Rook (2010, 2012) explicitly locates the old friends hypothesis in the context of Darwinian medicine, an approach that applies evolutionary theory to medical problems. Darwinian medicine is a framework that uses adaptationism as a heuristic principle to explain human health and disease. Proponents of Darwinian medicine assume that humans are genetically adapted to the hunter-gatherer environment of the Pleistocene and maladapted to modern environments of the industrialized Global North (mismatch hypothesis) which makes them susceptible to certain diseases (Méthot 2011, 78). Rook applies these theoretical principles to his discussion of immunological studies of host–parasite interactions. His aim is to show that and explain why the absence of helminths and other organisms plays an important role in the development of chronic inflammatory disorders in humans.

Rook (2010, 74) argues that helminths and other organisms “have been present, inevitably and continuously, from relatively early in the evolution of the immune system” and came to play an important role in immune regulation through a process of host–parasite coevolution. Due to their long association with humans, some species have evolved into commensals (i.e., old friends; Rook 2009). According to Rook, the human immune system has not been able to get rid of helminths in the parasite-rich environment of the Pleistocene, and over time, it came to depend on the presence of these organisms: “If we are thinking in a Darwinian way, we should be starting from the hypothesis that any organism that has been consistently present for a significant part of mammalian evolution might have been ‘written into’ the mammalian genome” (Rook 2010, 70). He takes the argument one step further and claims that the deprivation of certain organisms such as helminths contributes to the development of chronic inflammatory disorders, e.g., autoimmune diseases (Rook 2010, 2012). According to Rook, the proper functioning of our immune system depends on the presence of these organisms who have evolved from parasites into “friends” and “partners.” This so-called evolved dependence “refers to situations where an organism has become adapted to the presence of a partner through loss of genetic material, and can no longer function without that partner” (Rook 2010, 71). The old friends hypothesis would explain why autoimmune diseases are more common in Europe and North America than in countries of the Global South where helminth infections are prevalent (Rook 2012; WHO 2022).

#### Analysis of individuation practices

In Rook’s work, helminths are not referred to as ‘parasites’ and are not seen as invaders or opponents, but as friends and partners. Human–helminth interaction is conceptualized as a symbiotic relationship in which both partners benefit from the alliance. As in the review article discussed in the previous section, Rook’s individuation practices result in nested entities. Host and “parasite” are referred to as individual organisms but also conceptualized as a whole. While the focus in the article by Filyk and Osborne (2016) is on the intestinal ecosystem and interactions therein, Rook (2010) sees helminths as an integrated part of the human immune system. Thus, Rook’s emphasis is on the functioning of the host–parasite system as a whole. Although Rook does not explicitly use the concept of the holobiont, it applies to his notion of helminths and other organisms as integrated parts of the human immune system. The term ‘holobiont’ refers to a functionally, genetically, and spatially integrated biological unit that comprises a host and its symbiotic microbiota^13^ (Catania et al. 2017; Moran and Sloan 2015; Theis et al. 2016; Zilber-Rosenberg and Rosenberg 2008).

The question is whether the human–helminth holobiont as described by Rook also corresponds to a meta-scientific concept of biological individuality. I have argued that the multibiome is an ecological individual under the concept of ecological individuality. As I have already mentioned, the concept is versatile and can be applied to many different entities and levels of individuality. As the interactions between holobiont components are stronger than interactions between external components and holobiont, the holobiont is also an ecological individual under Huneman’s concept. But do strong concepts of individuality also apply in this case? Although helminths trigger immune responses in humans, Pradeu’s concept of immunological individuality would not adequately represent Rook’s conception of helminths as integrated parts of the human immune system. According to Rook, the helminth-induced immune response is what protects the host against autoimmune diseases and thus the helminth is not the human’s immunological antagonist but an integrated part of the immune system. If one were to understand the concept of immunological individuality as prescriptive, however, one could argue that Rook’s theorizing is flawed, and helminths should not be conceptualized as an integrated part of the human immune system.

Another possible candidate is evolutionary individuality, especially because Rook mentions the evolutionary dynamics that have led to the integration of helminths into the human immune system. As I have already mentioned, evolutionary individuality is usually associated with units of selection. Many authors argue that the holobiont is an entity targeted by natural selection (e.g., Dupré and O‘Malley 2009; Ereshefsky and Pedroso 2013, 2015; Godfrey-Smith 2009, 2013). Holobionts also exhibit other features associated with evolutionary individuality, e.g., they are integrated wholes, they are more or less delineated from their environments, and they have holobiont-level adaptive traits (Catania et al. 2017). They do not, however, reproduce on the level of the holobiont and they do not always form lineages with vertical transmission (from parents to offspring) of microbiota (Booth 2014; Godfrey-Smith 2009, 2013). Especially in the case of human–helminth systems, helminths do not spend their entire life cycle inside the host but are acquired from the environment. A hookworm (*Necator americanus*) larva, for example, hatches and grows in the soil and is able to penetrate the human skin when it has reached the third larval stage (Hotez et al. 2004). The adult worms lay eggs inside the human intestinal system and leave the body through feces (Hawdon and Hotez 1996). Therefore, the parasite is not directly transmitted from parents to offspring. For some authors, this point is crucial for characterizing holobionts as ecological communities rather than evolutionary individuals (e.g., Bourrat and Griffiths 2018; Skillings 2016). Others, however, see holobionts as evolutionary individuals although they do not reproduce as a unit. Ereshefsky and Pedroso (2015), for example, argue that instead of denying the holobiont’s individuality, we should rethink our concepts of evolutionary individuality. To sum up, the answer to the question whether the holobiont is an evolutionary individual depends on the criteria for evolutionary individuality. If one prioritizes functional integration over vertical transmission, the holobiont could be considered a biological individual in a strong sense. However, Rook mentions the evolutionary dynamics that have led to the integration of helminths into the human immune system, but he does not refer to the human–helminth system as a unit of selection. Whether the system as a whole is targeted by natural selection is not relevant for Rook’s argument and therefore the question whether the human-helminth system is an evolutionary individual is also irrelevant in the context of the old friends hypothesis.

## Individuation on different levels

In the previous section, I have discussed four studies on helminths and their vertebrate hosts. My analysis illustrates the plurality of individuation practices on different levels that correspond to different concepts of individuality and different notions of hosts and parasites (see Table 1). In the first study researchers investigated the cellular immune responses of sticklebacks infected with tapeworms. For this purpose, Scharsack and his collaborators individuated organisms and host cells. They conceptualized tapeworm and stickleback as individual organisms with an antagonistic relationship. The experiment showed that the tapeworm provoked an immune response in the stickleback. The individuation practices are best captured by a concept of immunological individuality. In this case, the stickleback can be understood as an immunological individual and the tapeworm is the invader that the stickleback’s immune system is trying to fight off. In the second case, the researchers tested whether two different stickleback populations vary with respect to immune phenotypes and evaluated whether there are underlying genetic differences. In this experimental study, stickleback and tapeworm were also conceptualized as antagonists. The scientists found that stickleback genes have an effect on the tapeworm’s phenotype and characterized the helminth as belonging to the extended phenotype of the host. I have argued that neither physiological nor evolutionary individuality account for the explanatory practices in question.

**Table 1:** Summary of the analysis of the four cases

Study	Research question	Levels of individuation	Conceptualization of host and parasite and their relationship	Concept of individuality
Scharsack et al. (2004) (experimental study)	What are the cellular immune responses of sticklebacks infected with tapeworms?	organism, cell	individual organisms, antagonistic relationship, host as an immunological individual, tapeworm as invader	immunological individuality
Weber et al. (2017) (experimental study)	Do the two different stickleback populations vary with respect to immune phenotypes and are there underlying genetic differences?	organism, population, extended phenotype	antagonistic relationship, but parasite as part of the host’s extended phenotype	no fitting meta-scientific concept
Filyk and Osborne (2016) (conceptual study)	How can we conceptualize the diversity of and interactions among biological entities of the mammalian intestinal system?	multibiome, organism	host as organism with a multibiome, parasites as constituents of the mammalian intestinal ecosystem	ecological individuality
Rook (2009, 2010, 2012) (conceptual studies)	Why are autoimmune diseases prevalent in industrialized countries?	holobiont, organism	host and parasite as constituents of the holobiont, parasite, parasites as “friends” and “partners”	ecological individuality, evolutionary individuality (if functional integration is prioritized over vertical transmission)

The third and fourth case represent individuation practices in scientific theorizing. Filyk and Osborne introduced the concept of the multibiome to account for the diversity of and interactions among biological entities of the mammalian intestinal system. Rook put forward the old friends hypothesis to explain the prevalence of autoimmune diseases in industrialized countries. In the paper by Filyk and Osborne, helminths are conceptualized as constituents of the mammalian intestinal ecosystem (the multibiome) while Rook sees them as constituents of a holobiont. Both the multibiome and the holobiont are biological wholes. The holobiont includes the host organism, while the multibiome comprises only the host’s microbiota and macrobiota. Both can be characterized as ecological individuals by applying the concept of ecological individuality. Whether the holobiont can additionally be characterized as an evolutionary individual depends on the respective criteria for evolutionary individuality. In all four studies individuation takes place on different levels or organization which results in nested biological systems. Scharsack et al. (2004), for example, individuate organisms and cells, while Filyk and Osborne (2016) individuate the host organism, the multibiome, its subsystems (e.g., macrobiome) and species. I have shown that some of the individuated systems are identical. Helminth and host, for example, are addressed as individual organisms in each of the four studies. Two studies (Sects. “The intestinal ecosystem” and “”Helminths as old friends) additionally individuate the host–parasite system as a whole. My analysis suggests that the partitioning frame, i.e., the criteria for identifying and individuating biological entities (Winther 2006) is similar in all four studies. In none of the studies the researchers individuated entities like molecules, organs, or genes. This is probably owed to the fact that the scientists work in similar fields and share the same theoretical perspective (Winther 2006). However, the scientists’ emphasis differs with respect to the levels of organization. Depending on the research question and the explanandum, they concentrate either on cells, individual organisms, populations, systems that comprise members of different species, or the host–parasite system as a whole (Table 1).

The results of my analysis suggest that individuation practices are, to a certain extent, linked to research fields in the sense that scientists seem to draw from the same repertoire of entities (e.g., cells, organism, holobiont), even when following different projects within one field. Individuation practices could also depend on the kinds of organisms that are studied by the scientists. The variety of levels of organization and the different concepts of helminths and vertebrate hosts (e.g., helminth as invader vs. friend) and their relationship (host and parasite as individual organisms in an antagonistic relationship vs. helminths as integrated parts of the host’s immune system), however, suggest that the kind of organism is not the main factor. Instead, the researchers’ focus on a certain level of organization is guided by their research question (see Love 2018). Thus, the focus on different levels of organization corresponds to different epistemic aims, purposes and contexts of investigation (Love and Brigandt 2017; Reydon 2021; Waters 2018). These purposes, however, are not adequately captured by general categories such as explaining, predicting, and manipulating, but are more fine-grained and local (see Waters 2018). In the studies discussed in the previous section, scientist individuated units to explain specific aspects about host–parasite systems. The study by Weber and his collaborators, for example, explains why tapeworms grow larger in one population of sticklebacks than in another population. The old friends hypothesis, on the other hand, is a possible explanation for the fact that very few people in countries of the Global South suffer from allergies and other autoimmune diseases while these diseases are prevalent in countries of the Global North. Therefore, explanations of different phenomena in similar organisms can require a focus on different levels of organization and depend on the explanandum. It is also important to note that scientist sometimes use different concepts for individuating entities during different stages of their work. Weber and his team, for example, conceptualized stickleback and helminth as individual organisms that represent populations for the purpose of experimentally manipulating and analyzing them. For explaining their interaction, however, they used the concept of the extended phenotype.

I have shown that individuation practices do not necessarily map neatly onto established meta-scientific concepts of individuality. The concept of ecological individuality developed by Huneman is a formal concept that requires a theoretical basis to empirically determine individuality, is versatile and could potentially be used to account for individuation practices in all four studies. Stronger concepts of individuality like physiological or evolutionary individuality, however, do not apply to at least two of the studies (Sects. “How host evolution affects parasites” and “The intestinal ecosystem”). In the first study, the concept of immunological individuality captures Scharsack and collaborators’ individuation practices quite well while there is no established meta-scientific concept of strong biological individuality that adequately captures explanatory practices discussed in section “How host evolution affects parasites”. My analysis suggests that in some cases theory (concepts of individuality) and practice (individuation) are well matched. However, as we have seen, this is not always the case. These findings and the fact that none of the researchers discuss concepts of individuality in their publications could mean that the scientist consider their concept of individuality to be clear and do not see the need to explicitly mention it or, more likely, they do not follow a top-down strategy to individuate entities, meaning that their practices are not strictly guided by concepts of individuality. In the following section, I discuss this question and argue that concepts of individuality should be understood as models that do not necessarily represent all instances of scientific practice.

## Concepts of individuality as models

Although most scientists do not seem to have a clear-cut answer to the question “What entities are biological individuals?”, they are able to successfully individuate living systems for their respective purposes (Kovaka 2015). A fundamental theory of individuality is thus unnecessary and maybe even unwarranted for individuation practices (Love 2018, 185). Often, scientists use lower-level concepts like ‘organism’ or ‘holobiont’ as tools to individuate biological systems (Catania et al. 2017; Waters 2018) and their individuation practices are guided by their research questions and explananda (Love 2018) as well as practical considerations. Kovaka (2015) argues against the widespread conviction that individuation practices depend on theories of biological individuality and proposes a “sensitivity account” to characterize the relationship between theory and practice. She argues that “biologists do not need to know what a biological individual is in order to do good empirical work, but which objects they count as individuals does affect their thinking about biological processes” (Kovaka 2015, 1095). In turn, empirical findings can influence the way scientists and science studies scholars think about biological individuality and alter individuation criteria.

**Fig. 1:**
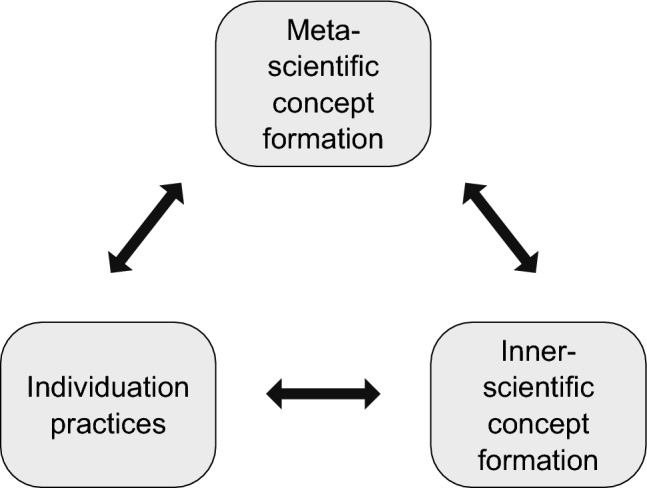
Relationships between individuation practices and concept formation

Scientific practices are informed by theoretical concepts and vice versa; the relationship between concepts of individuality and individuation practices is one of “mutual dependence” (Kovaka 2015, 1102). A relationship of mutual dependence explains the fact that established concepts of individuality do not capture the practices equally well. A mismatch between theoretical and practical considerations can appear because individuation of biological systems is not entirely governed by concepts and theories, nor are concepts of biological individuality entirely based on scientific practices. Similarly, the relationship between inner-scientific and meta-scientific concepts of individuality is one of reciprocal dependence. It is also possible that meta-scientific concepts are strongly influenced by individuation practices and vice versa. I thus propose to conceptualize the relationship between theory and practice as a triangle of relationships of reciprocal dependence between individuation practices, inner-scientific concept formation and meta-scientific concept formation (Fig. 1).

Figure 1 illustrates the reciprocal dependence between individuation practices and inner-scientific concept formation. For example, scientific findings have led to the establishment of the concept of the holobiont and in turn, the concept of the holobiont changes the way scientists view and conceptualize certain biological systems. But there is also a reciprocal relationship between inner-scientific and meta-scientific concepts of individuality. The application of the concept of the holobiont might change the way science studies scholars define evolutionary individuality. On the other hand, the discussions on the evolutionary individuality of the holobiont might also lead to a revision of the concept of the holobiont. Meta-scientific concepts of individuality and scientific practices are also in a direct reciprocal relationship with each other, as the following example of Love’s (2018) study of individuation practices in developmental biology shows.

As I have argued in the previous sections, some individuation practices cannot be adequately captured by established meta-scientific accounts of biological individuality. Love’s (2018) study of individuation practices in developmental biology has yielded similar results. He argues that there is a mismatch between theory and practice because “developmental biologists do not rely on a theory of biological individuality based on natural selection to track developing embryos and their component parts.” According to Love (2018), their research is not governed by a fundamental theory, but instead guided by structured problems. He claims that “the appeal to fundamental theory from evolutionary biology [as in Clarke’s (2010, 2013) and Godfrey-Smith’s (2009, 2013) accounts] gets it wrong” (Love 2018, 185). He thus suggests that philosophers should focus their attention on individuation practices to generate accounts of individuality that describe and explain scientific practices adequately (Love 2018, 188). However, Love does not discuss other concepts of biological individuality (e.g., developmental individuality, physiological individuality) to examine if they capture the practices in developmental biology better than concepts of evolutionary individuality.

I agree that a bottom-up approach from individuation practices to meta-scientific concept formation would yield accounts of biological individuality that are more in line with scientific practice. Pradeu’s concept of immunological individuality, for example, resulted from philosophical inquiry that is strongly practice-oriented. My analysis of Scharsack et al.’s practices suggests that Pradeu’s insights into scientific practices and collaboration with immunologists yielded a concept of individuality that accounts for certain individuation practices in immunological research. However, his account is rather local and does not capture other practices. I would not say, however, that Pradeu’s appeal to immunological theory gets it wrong in the other cases (e.g., Rook’s work on the old friends hypothesis). Instead, I propose that concepts of individuality should be understood as different models. In this context, models are simplified accounts that help scientists and philosophers describe, understand, explain and gain access to biological phenomena (see Bailer-Jones 2002; Morgan 2001). As models, concepts of individuality can be abstract and general (e.g., Huneman’s concept of ecological individuality) or specific (e.g., Pradeu’s concept of immunological individuality) representations of scientific practice.^14^ More specific concepts are local in the sense that they only represent certain aspects of scientific practice or practices revolving around certain research questions, but they rely on rather concrete individuation criteria (e.g., immunogenicity). General concepts provide very general individuation criteria (e.g., the strength of interactions) and thus represent a larger class of individuation practices in various fields and contexts.

As models, concepts of individuality are flawed by definition. Models are abstract, idealize, simplify or omit certain aspects of the target phenomena (Frigg et al. 2020; Giere 2004; Morrison and Morgan 1991). Thus, they do not represent all instances of target phenomena equally well. Instead, they are tools for different purposes like manipulation, prediction, or explanation (Knuuttila 2011; Parker 2010)﻿. At the same time, representations like models influence the way we perceive the world (see Winther 2020).^15^ Although concepts of individuality do not perfectly represent scientific practices, they are still useful tools that help science studies scholars describe and explain scientific practices and at the same time inform the way scientist perceive their research objects. Concepts of biological individuality are not constructed in strict top-down or bottom-up approaches. Instead, they are situated somewhere between theory and practice because both empirical findings and theory enter into the modeling process (Morrison and Morgan 1999). While some concepts of biological individuality are located further toward the theoretical pole of the theory–practice continuum, others are more practice-based. Thus, a modeling account of biological individuality is in line with the relationship of reciprocal dependence between inner-scientific and meta-scientific concepts of individuality and scientific practices as represented in Fig. 1.

My analysis suggests that similar organisms and systems are conceptualized and individuated differently in different research contexts which suggests a pluralistic view of biological individuality. A modeling account of biological individuality is compatible with pluralism of different kinds of biological individuality such as developmental individuality, evolutionary individuality and physiological individuality (see DiFrisco 2019). In some cases it might even be adequate to represent individuation practices with more than one model. Considering the diversity of scientific practices and the complexity of biological entities, it seems difficult to capture all aspects of this variety with only one model of biological individuality (see Kovaka 2015). A modeling account of biological individuality is also compatible with a pluralistic perspective on the question whether concepts of individuality should be normative or descriptive. While some concepts of individuality adequately describe certain aspects of scientific practice in certain contexts, other concepts of individuality are normative because they guide scientific practices. For example, concepts of evolutionary individuality can guide the choice of evolutionary models or provide a coherent categorization of biological entities (Sterner 2015, 614). Concepts of biological individuality can also be normative when they are applied in ethical discussions (e.g., discussions of intrinsic value of or moral obligation toward biological entities; see Millstein 2018). However, most concepts are probably located somewhere in between the two poles of the descriptive-normative continuum.

With the view of concepts of individuality as models, the question is no longer whether the model “gets it right” or whether it is true, but whether it is adequate for the purpose in question (Parker 2010, 2020). Therefore, in the case described by Love (2018) the conclusion would be that concepts of individuality that are based on evolutionary theory are not adequate for representing or guiding the individuation practices applied by developmental biologists. This does not necessarily mean that there is something wrong with evolutionary concepts of biological individuality, but maybe only that these models are not the right tools in this context. A concept of developmental individuality would probably be more suitable in this case. Of course, models can and should be revised on the basis of empirical findings, but in this case, it seems more appropriate to find a different model or create a new one. Even if suitable models (such as concepts of developmental individuality) already exist, it is unlikely that they represent all aspects of scientific practice perfectly. Thus, my answer to the question ‘Do scientists need to adjust their practices to bring them in line with existing concepts or should the concepts of biological individuality be revised?’ is ‘both and neither.’ Depending on the context, it might be appropriate to revise the model while in other cases scientists might want to rethink their practices. In the case of Weber et al.’s (2017) study, the conceptualization of the parasite as part of the host’s extended phenotype unnecessarily complicates things. Instead, it would also have been possible to conceptualize host and parasite as physiological individuals with intertwined evolutionary trajectories. One could also argue that the extended phenotype is not a biological individual and should therefore not be conceptualized in terms of biological individuality. In other cases, if the model is good enough, it may also be justified to use a model that is flawed as long as one is aware of the model’s limitations. In the case of the human–helminth holobiont, for example, one could argue that existing concepts of evolutionary individuality account for the practices in question, even though holobionts do not exhibit all features associated with evolutionary individuality.

## Footnotes

1 Here, ‘inner-scientific’ refers to concepts of individuality that are constructed by scientists. Meta-scientific concepts of individuality are concepts that are constructed by science studies scholars (e.g., philosophers and historians of science).

2 This point is rarely made explicit, but is implied by certain formulations, e.g., that concepts of individuality are “applied to” a biological system of interest. Ellen Clarke (2013, 413) states that biologists “need some generalized guidelines that tell them what to look for when deciding if some unit should be counted.”

3 I take the notion of ‘scientific practices’ to include all types of scientific activities such as experimenting, conceptualizing, explaining and theorizing.

4 Here, I am not referring to concepts of biological individuality, but to lower-level concepts such as ‘organism,’ ‘extended phenotype’ and ‘multibiome’ that are used as tools to individuate biological systems.

5 I chose these cases mainly for epistemic reasons, i.e., because together they represent the plurality of individuation practices within one field. It was also important to me to discuss experimental as well as conceptual practices to portray a broad spectrum of scientific practices within one field. Pragmatic criteria played an important role in the choice of the cases. For example, I worked with Jörn Scharsack on an experiment on Stickleback and tapeworms at the University of Münster where I was introduced to this experimental system. The collaboration with him and his team sparked my interest in the topic of biological individuality and host–parasite systems. Scientific impact did not play a role in the choice of the cases. It is true that I chose these cases to make a certain point and that my choice was biased and guided by my theoretical assumptions, but this does not mean that my analysis misrepresents the variety of scientific practices in studies of host–parasite systems. For detailed discussions of cherry-picking and theory-ladenness of case work in history and philosophy of science see, e.g., Schickore (2011), Currie (2015), Kinzel (2015), Scholl and Räz (2016).

6 Flow cytometry is a technique that scientists apply to determine the characteristics of cells (e.g., size, surface structure).

7 Granulocytes and lymphocytes are specific types of white blood cells.

8 The procercoid stage is the first larval stage of the tapeworm.

9 The plerocercoid stage is the second larval stage of the tapeworm.

10 Sticklebacks in 50 Lakes on Vancouver Island were monitored over a time period of 10 years (Weber et al. 2017).

11 The name of the hypothesis is derived from Lewis Carroll’s novel *Through the Looking-Glass and What Alice found There*. In the novel, the Red Queen says to Alice, “Now, here, you see, it takes all the running you can do, to keep in the same place.”

12 Surely, there are simpler ways of explaining the study results that do not rely on the concept of the extended phenotype. However, at this point I describe the scientists’ practices (what they did) without judging them (i.e., discussing what the authors should or could have done instead) and analyze them in relation to concepts of biological individuality. In the final section, I briefly discuss the scientists’ interpretation critically.

13 Here, I use ‘microbiota’ as a broad concept that refers to an assemblage of bacteria, archaea, viruses, protists, fungi, and helminths.

14 The distinction between specific and general concepts should not be understood as a dichotomy but rather as two poles of a continuum.

15 Blackwell and Engelhardt (2002, 54) present a similar argument and claim that representations like diagrams affect mental representations of phenomena. The London Underground map, for example, has changed people’s “mental map” of distances across London.
